# Supplementary material for: Whole-Transcriptome Sequencing Analyses of Nuclear Antixoxidant-1 in Endothelial Cells: Role in Inflammation and Atherosclerosis
Source: Cells. 2022 Sep 18;11(18):2919. doi: 10.3390/cells11182919 (PMC9496719; doi:10.3390/cells11182919)
Supplement: Supplementary file 1 [file cells-11-02919-s001.zip › cells-1822705-Supplementary figures.pdf]

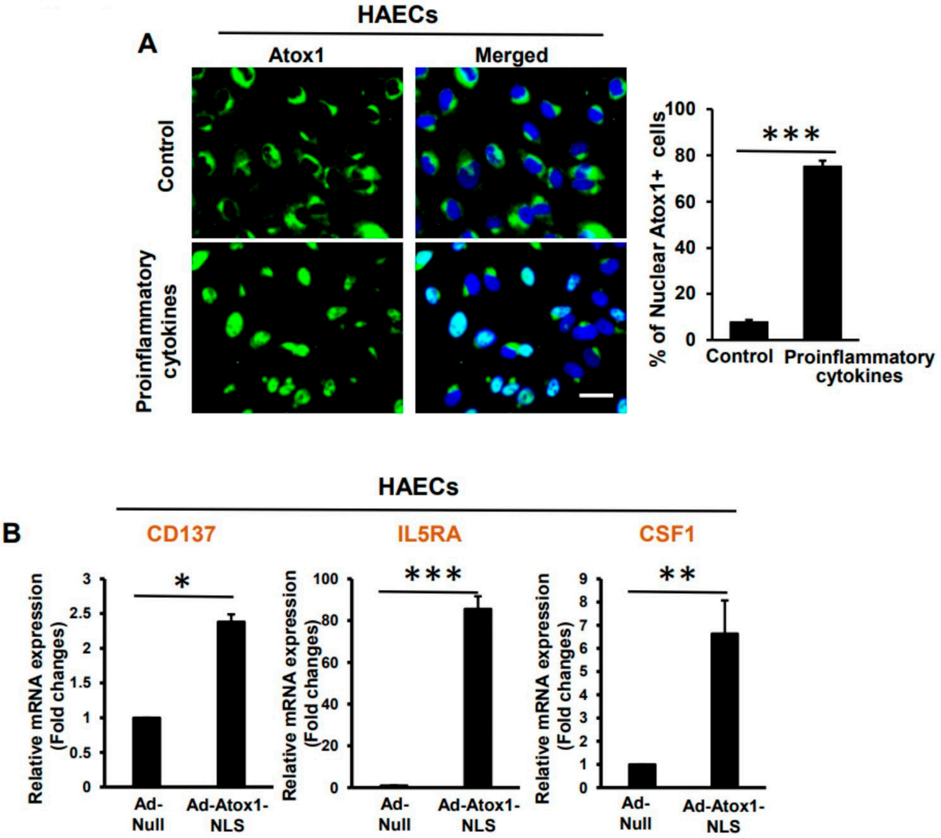

Figure S1. (A) Immunofluorescence staining of Atox1 in HAECs. HAECs stimulated with proinflammatory cytokine cocktail (TNF-α [10 ng/mL], IL-1β [10 ng/mL], and IL-6 [10 ng/mL] for 1h were immunostained for Atox1 using anti-Atox1 antibody or nuclear marker DAPI, as described in Figure 1. Percentage of Atox1+ cells in nucleus was shown in right (n = 3). Scale: 20   m. (**B**) CD137, IL15RA and CSF1 as Atox1 targets in HAECs. HAECs were transfected with Ad-Atox1-NLS or Ad-null for 48 hrs. Cells were harvested and CD137 or IL5RA or CSF1 mRNA expression measured (n=3). ***P<0.001, **P<0.01 and *P<0.05.

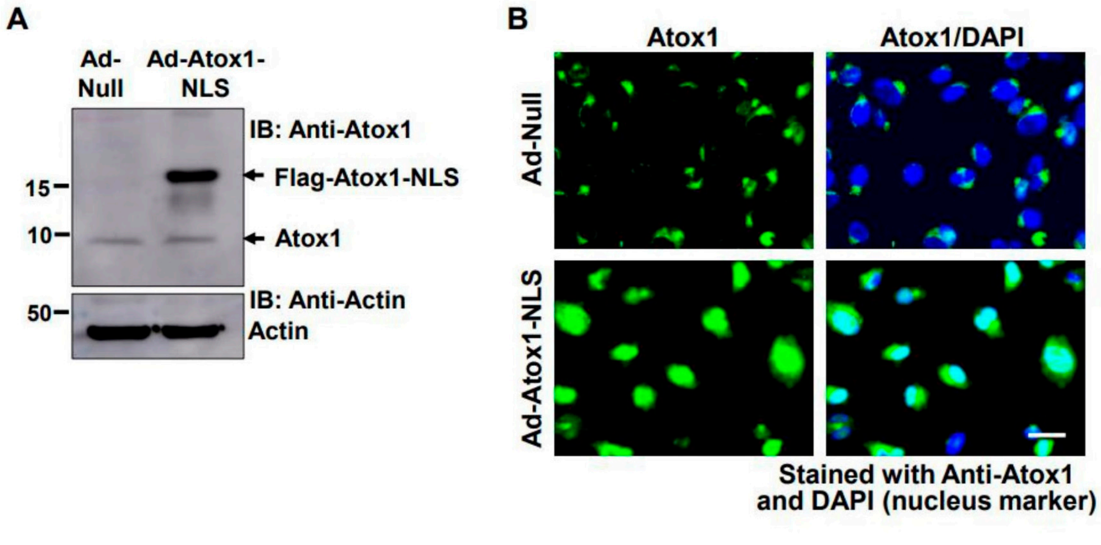

Figure S2. A. Atox1 expression in ECs transfected with Ad-Atox1-NLS. HUVECs were transfected with Ad-Null or Ad-Atox1-NLS and total cell lysate were subjected to western blotting with anti-Atox1 or anti-actin (n=3). **B.** Subcellular localization of Atox1 in Ad-Atox1-NLS or Ad-Null transfected HUVECs. HUVECs were transfected with Atox1-NLS or Ad-null immunolabeled with antibodies for Atox1 (green) and nuclear marker, 4′,6-diamidino-2-phenylindole (DAPI) (Blue), Scale: 20 m. (n=3).

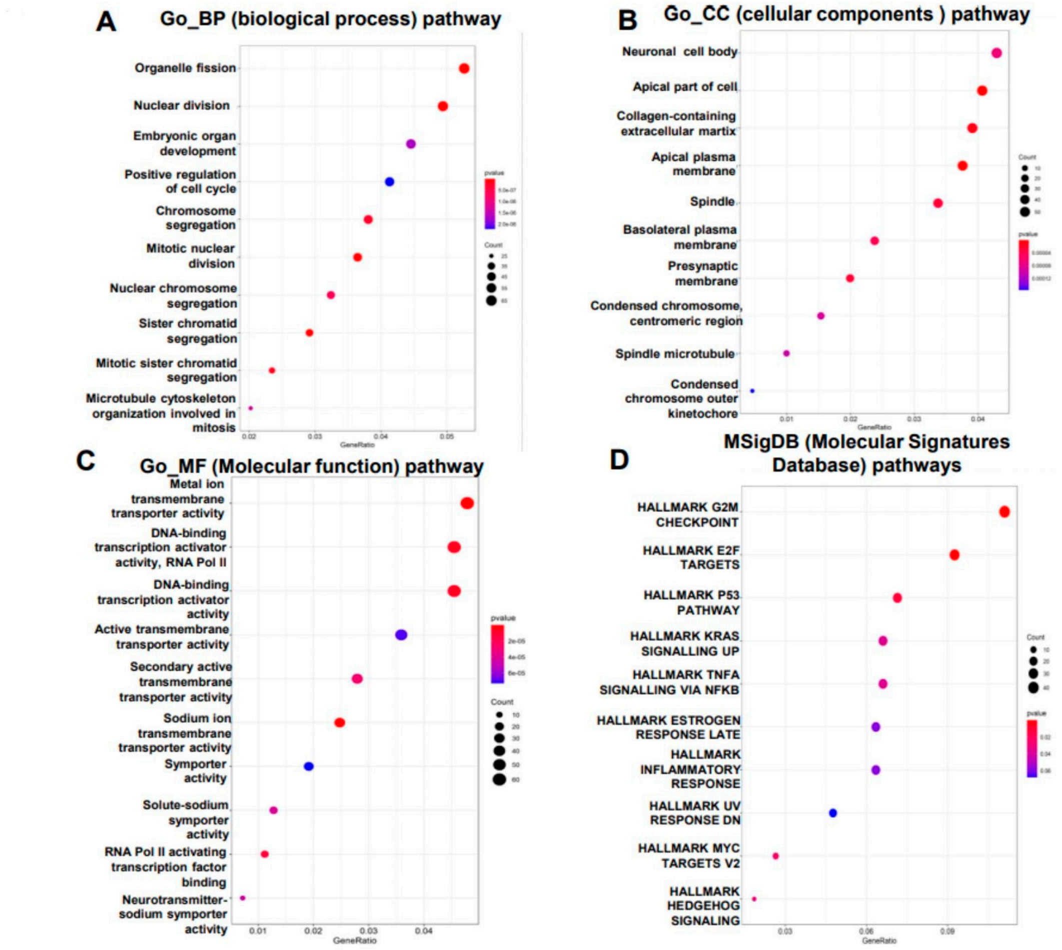

Figure S3. Analysis of RNA-Seq and ChIP-Seq data. A-D. Gene ontology (GO) and MSigDB pathway enrichment analysis of 1387 DEGs identified by RNA-Seq and ChIP-Seq (corresponding to Figure **3A**). Top 10 enriched GO terms of biological processes (**A**), cellular components (**B**), molecular function (**C**) and the Molecular Signatures Database (**D**) ranked by the number of DEGs in the GO term/pathway are shown.

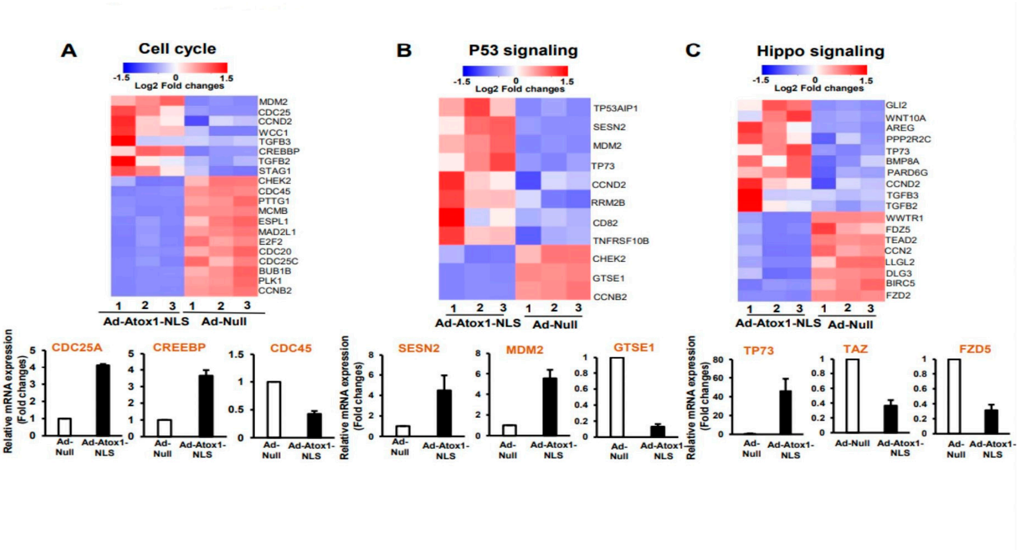

Figure S4. Regulation of genes from KEGG pathways enriched by Atox1-NLS. KEGG pathway enrichment analysis of 1387 DEGs identified by RNA-Seq and ChIP-Seq (corresponding to Figure 3A). **A-C**. Top: Heatmap showing the expression levels of DEGs in RNA-Seq belonging to the cell cycle (**A**), P53 signaling (**B**) and Hippo signaling (**C**) pathways in ECs treated with Atox1-NLS. Color scale depicts range of log2 fold changes in gene expression. Bottom: differentially expressed gene in this pathway was validated by quantitative RT-PCR (n=3).

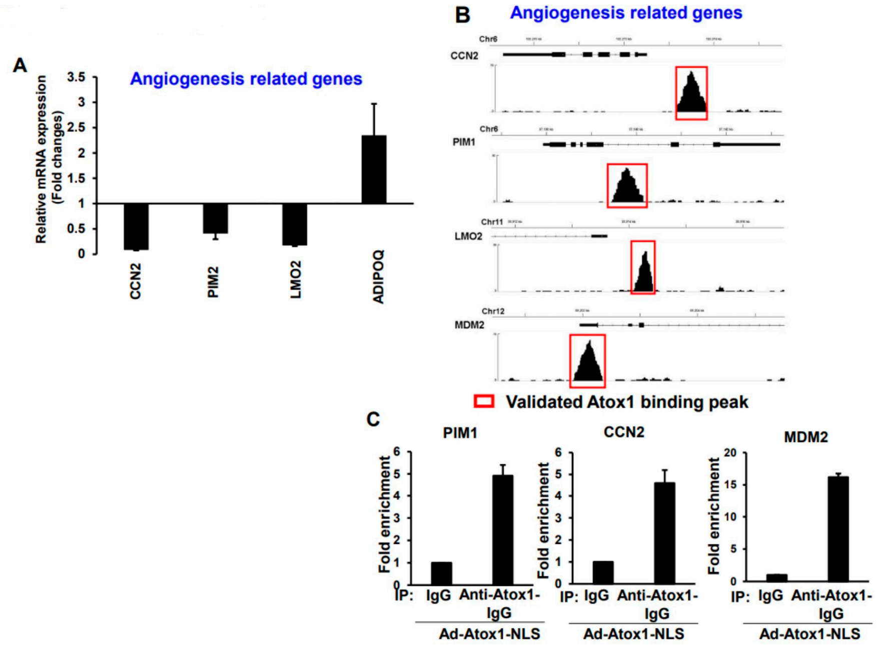

Figure S5. Validation of Atox1 binding peaks. A. Subset of the Atox1 regulated gene targets was validated by quantitative RT-PCR (n=3). **B**. Representative tracks from ChIP-Seq results show the locations of Atox1 binding peaks proximal to the TSS of Atox1 targeted genes. **C**. HUVEC cells were transfected with Atox1-NLS for 48 hrs and subject to ChIP using Atox1 monoclonal Ab and mouse IgG. The enrichment of Atox1 binding peaks were determined by qPCR.

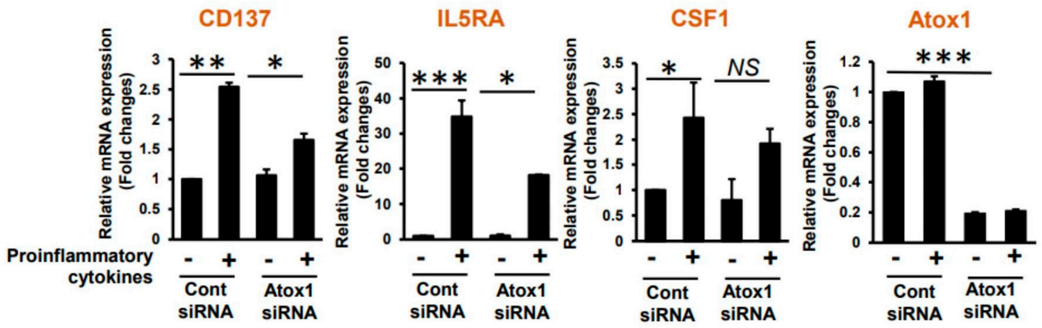

Figure S6. Role of Atox1 in CD137 or IL5RA or CSF1 mRNA expression in ECs treated with proinflammatory cytokines: Cells transfected with Ad-Atox1-NLS or Ad-Null were treated with proinflammatory cytokine cocktail (TNF-α [10 ng/mL], IL-1β [10 ng/mL], and IL-6 [10 ng/mL] for 24h in the presence of Con siRNA or Atox1 siRNA. CD137 or IL5RA or CSF1 mRNA expression were assessed by quantitative PCR (n=3). ***P<0.001, **P<0.01 and *P<0.05.
