# Supplementary material for: Whole-Transcriptome Sequencing Analyses of Nuclear Antixoxidant-1 in Endothelial Cells: Role in Inflammation and Atherosclerosis
Source: Cells. 2022 Sep 18;11(18):2919. doi: 10.3390/cells11182919 (PMC9496719; doi:10.3390/cells11182919)
Supplement: Supplementary file 1 [file cells-11-02919-s001.zip › Supplemental Table 8 and 9 - 08-27-2022.pdf]

Supplemental Tables (Tables S8 and S9)

Whole-transcriptome sequencing analyses of Nuclear Antixoxidant-1 in Endothelial Cells: Role in inflammation and Atherosclerosis

#Varadarajan Sudhahar [1,2,6], #Yang Shi[4], Jack H. Kaplan, Masuko Ushio-Fukai[1,3],

*Tohru Fukai,[1,2,6]

[1]Vascular Biology Center, [2]Department of Pharmacology and Toxicology, [3]Department of Medicine (Cardiology), and [4]Deptartment of Population Health Science, Medical College of Georgia at Augusta University, Augusta, GA; [5]Department of Biochemistry and Molecular Genetics, University of Illinois College of Medicine, Chicago, IL, [6]Charlie Norwood Veterans Affairs Medical Center, Augusta, GA

# equally contributed first author

*Address correspondence to:

Tohru Fukai, MD, PhD
Vascular Biology Center
Dept of Pharmacology & Toxicology
Medical College of Georgia
at Augusta University
1460 Laney-Walker Blvd,
Augusta, GA 30912, USA
Email: tfukai@augusta.edu

Supplementary Table S8: Primer sequences utilized for ChIP–qPCR experiment

Target gene	Position of binding site in genome	Primer sequence (5'-3')
ADRB1	Chr10:115801066-115801354	Forward- TTCCGGTAACACCACTTGACA
		Reverse- GGGTGAGGCATAGGGAATGT
CCN2	Chr6:132273291-132273700	Forward- CCCAAGGAACATGCAGCCT
		Reverse- CACCCAGGGAGAATAAAGCC
CSF1	Chr1:110451188-110451436	Forward- ACCACTCCTTTGGGGAAAGTC
		Reverse- GGCCTGAAGACAGTAAGTGC
IL5RA	Chr3: 3133776-3134106	Forward- GTTGGCTCCACTCACTCCAG
		Reverse- TTCTTCTAATGCTATCTTTCCCCAC
PIM1	Chr11:37140611- 37140991	Forward- CCACTCTCCTTAGCCCAGAG
		Reverse- GGCTATACACTCGGGTCCCT
MDM2	Chr12: 69201981-69202226	Forward- TGTGTGTCGGAAAGATGGAGC
		Reverse- CAGGATCTCGGTCAGAGGGG
CD137	Chr1:8000546-8000923	Forward- ATCACTCTTCCCCCTTTAGCTG
		Reverse- TTTGCTCCCTTTGCCCATTTC

Supplementary Table S9: Primers used for qPCR

Gene target	Primer sequence (5’-3’)
CDC25A - Forward	TCTGGACAGCTCCTCTCGTCAT
CDC25A - Reverse	ACTTCCAGGTGGAGACTCCTCT
CREBBP - Forward	AGTAACGGCACAGCCTCTCAGT
CREBBP - Reverse	CCTGTCGATACAGTGCTTCTAGG
CDC45 - Forward	TGGATGCTGTCCAAGGACCTGA
CDC45 - Reverse	CAGGACACCAACATCAGTCACG
TRAF1 - Forward	CGATGGCACTTTCCTGTGGAAG
TRAF1 - Reverse	TACAGCCGCAGGCACAACTTGT
CSF1 - Forward	TGAGACACCTCTCCAGTTGCTG
CSF1 - Reverse	GCAATCAGGCTTGGTCACCACA
MAP3K14 - Forward	GGAATACCTCCACTCACGAAGG
MAP3K14 - Reverse	CTGTGAGCAAGGACTTTCCCAG
SESN2 - Forward	AGATGGAGAGCCGCTTTGAGCT
SESN2 - Reverse	CCGAGTGAAGTCCTCATATCCG
MDM2 - Forward	TGTTTGGCGTGCCAAGCTTCTC
MDM2 - Reverse	CACAGATGTACCTGAGTCCGATG
GTSE1 - Forward	CTCTACCAGCAATCTCGCAAGG
GTSE1 - Reverse	GACTTGCTGATGTTTGACAGAGG
TP73 - Forward	CATGGAGACGAGGACACGTACT
TP73 - Reverse	TGCCGATAGGAGTCCACCAGTG
TAZ - Forward	GAGGACTTCCTCAGCAATGTGG
TAZ - Reverse	CGTTTGTTCCTGGAAGACAGTCA
FZD5 - Forward	TGGAACGCTTCCGCTATCCTGA
FZD5 - Reverse	GGTCTCGTAGTGGATGTGGTTG
ADRB1 - Forward	TTCCTGCCCATCCTCATGCACT
ADRB1 - Reverse	GTAGAAGGAGACTACGGACGAG
CCN2 - Forward	CTTGCGAAGCTGACCTGGAAGA
CCN2 - Reverse	CCGTCGGTACATACTCCACAGA
PIM1 - Forward	TCTACTCAGGCATCCGCGTCTC
PIM1 - Reverse	CTTCAGCAGGACCACTTCCATG
LMO2 - Forward	GCGCCTCTACTACAAACTGGGC
LMO2 - Reverse	CTCATAGGCACGAATCCGCTTG
ADIPOQ - Forward	CAGGCCGTGATGGCAGAGATG
ADIPOQ - Reverse	GGTTTCACCGATGTCTCCCTTAG
ERBB3 - Forward	CTATGAGGCGATACTTGGAACGG
ERBB3 - Reverse	GCACAGTTCCAAAGACACCCGA
CD137 - Forward	TCTTCCTCACGCTCCGTTTCTC
CD137 - Reverse	TGGAAATCGGCAGCTACAGCCA
DUSP16 - Forward	TGCCAGCGAGATGTCCTCAACA
DUSP16 - Reverse	ACACGCAGGAAATGAGACTCGG
TNFSF11 - Forward	GCCTTTCAAGGAGCTGTGCAAAA
TNFSF11 - Reverse	GAGCAAAAGGCTGAGCTTCAAGC

MAFA - Forward	GCTTCAGCAAGGAGGAGGTCAT
MAFA - Reverse	TCTGGAGTTGGCACTTCTCGCT
IL5RA - Forward	TGACTGGCTTGCGGTGCTTGTT
IL5RA - Reverse	CTGCTGTGACATTCAGTGGAGG
Atox1 - Forward	CTTCTTGTTGGGCAGGTCAATG
Atox1 - Reverse	CAGTCATGCCGAAGCACGAG
